# Changes in the Metabolome in Response to Low-Dose Exposure to Environmental Chemicals Used in Personal Care Products during Different Windows of Susceptibility

**DOI:** 10.1371/journal.pone.0159919

**Published:** 2016-07-28

**Authors:** Sander M. Houten, Jia Chen, Fiorella Belpoggi, Fabiana Manservisi, Alberto Sánchez-Guijo, Stefan A. Wudy, Susan L. Teitelbaum

**Affiliations:** 1 Department of Genetics and Genomic Sciences, Icahn School of Medicine at Mount Sinai, New York, NY, United States of America; 2 Icahn Institute for Genomics and Multiscale Biology, Icahn School of Medicine at Mount Sinai, New York, NY, United States of America; 3 Department of Preventive Medicine, Icahn School of Medicine at Mount Sinai, New York, NY, United States of America; 4 Department of Pediatrics, Icahn School of Medicine at Mount Sinai, New York, NY, United States of America; 5 Department of Oncological Sciences, Icahn School of Medicine at Mount Sinai, New York, NY, United States of America; 6 Department of Medicine, Hematology, and Medical Oncology, Icahn School of Medicine at Mount Sinai, New York, NY, United States of America; 7 Cesare Maltoni Cancer Research Centre, Ramazzini Institute, Bentivoglio (Bologna), Italy; 8 Steroid Research and Mass Spectrometry Unit, Division of Pediatric Endocrinology and Diabetology, Center of Child and Adolescent Medicine, Justus Liebig University, 35392, Giessen, Germany; Utah State University, UNITED STATES

## Abstract

The consequences of ubiquitous exposure to environmental chemicals remain poorly defined. Non-targeted metabolomic profiling is an emerging method to identify biomarkers of the physiological response to such exposures. We investigated the effect of three commonly used ingredients in personal care products, diethyl phthalate (DEP), methylparaben (MPB) and triclosan (TCS), on the blood metabolome of female Sprague-Dawley rats. Animals were treated with low levels of these chemicals comparable to human exposures during prepubertal and pubertal windows as well as chronically from birth to adulthood. Non-targeted metabolomic profiling revealed that most of the variation in the metabolites was associated with developmental stage. The low-dose exposure to DEP, MPB and TCS had a relatively small, but detectable impact on the metabolome. Multiple metabolites that were affected by chemical exposure belonged to the same biochemical pathways including phenol sulfonation and metabolism of pyruvate, lyso-plasmalogens, unsaturated fatty acids and serotonin. Changes in phenol sulfonation and pyruvate metabolism were most pronounced in rats exposed to DEP during the prepubertal period. Our metabolomics analysis demonstrates that human level exposure to personal care product ingredients has detectable effects on the rat metabolome. We highlight specific pathways such as sulfonation that warrant further study.

## Introduction

Public concerns over the effects of ubiquitous exposure to personal care products have increased [1]. This concern has resulted in legislation restricting the use of many chemicals with potential negative health effects in cosmetics and other personal care products. These ingredients are included for multiple purposes such as anti-microbial activity (e.g. triclosan), product preservative (e.g. parabens), or fragrance stabilization (e.g. diethyl phthalate). Since these chemicals can have unintended biological activities, a more thorough understanding of their system-wide effects is needed.

Metabolomics is the comprehensive and systematic identification and quantification of small molecules (metabolites) in a biological sample [2]. The value of metabolomics lies in the fact that it profiles the biological processes that are considered most proximal to a specific phenotype or disease, which enhances our ability to discover causal associations [3]. Moreover the metabolome is not only determined by an individual’s genome, it is also reflective of everyday life or specific toxicant exposures and the body’s response to these exposures. Specific consideration of the metabolic response to environmental exposures of chemicals such as personal care product ingredients has been referred to as environmental metabolomics [4].

In comparison with other “-omics” techniques, the application of metabolomics has only recently begun to gain momentum and has slowly incorporated environmental exposures into their analyses. The slow progress in metabolomics was primarily the result of technical challenges such as the wide range in concentrations and physicochemical properties of metabolites. Metabolomics has been successfully applied in many studies ranging from biomarker discovery to genome wide association studies [5–7]. Several metabolomics studies addressed exposure to environmental chemicals such as di(2-ethylhexyl)phthalate, bisphenol A and arsenic [8–13].

Diethyl phthalate (DEP) is the diethyl ester of phthalate, an aromatic compound. DEP is often added as a stabilizer in personal care products and flexible plastics. DEP has been found to exhibit anti-androgenic activity [14], but only weak or no estrogenic activity [15]. DEP exposure has been associated with increased breast cancer risk [16, 17] and increased body size in children [18]. Methylparaben (MPB or methylparahydroxybenzoate) is often used as a preservative in personal care products, cosmetics, pharmaceuticals and in the processing of foods and beverages. Parabens have been found to have estrogenic activity [19]. Triclosan (TCS) is a chlorinated aromatic compound that has antibacterial and antifungal properties and is added to personal care as well as household products. TCS has been shown to act as an androgen [14], alter female reproductive development and enhance the effects of estrogen [20], decrease fecundity [21] as well as act as a thyroid toxicant [22]. These three common personal care product ingredients have not been investigated using environmental metabolomics approach.

Toxicological studies on environmental chemicals often employ animal models with treatment doses much higher than human exposure. In addition, these studies usually do not take into account the intrinsic differences in biological response to environmental chemicals at distinct developmental stages or so-called “windows of susceptibility”. We designed an animal experiment that simultaneously addresses these two issues. We have recently reported that in rats there is a consistently positive linear relationship between oral dose of DEP, MPB and TCS and urinary excretion of their metabolites, which enabled us to identify oral doses of these chemicals that yield levels of urinary metabolites typically observed in humans [23]. Using the same Sprague-Dawley rat as our model system, we investigated the effect of DEP, MPB, TCS, and their mixture on the blood metabolome in 3 windows of exposure, i.e. prepubertal, pubertal and chronic (birth to adulthood). We employed doses comparable to the US population exposure, which are 1,000–10,000 fold lower than the no-observed-adverse-effect-level (NOAEL) and much lower than most doses used in published studies [23]. Exposure at such low doses is unlikely to result in toxicity, phenotypic changes or disease outcome. Instead we used non-targeted metabolomics profiling to detect sensitive biomarkers reflecting exposure. Our results revealed that human-level exposure to commonly used ingredients in personal care products has significant effects on the metabolome and produces signature changes depending upon chemical used and the window of susceptibility.

## Materials and Methods

### Animal Experiment

**Fig 1:**
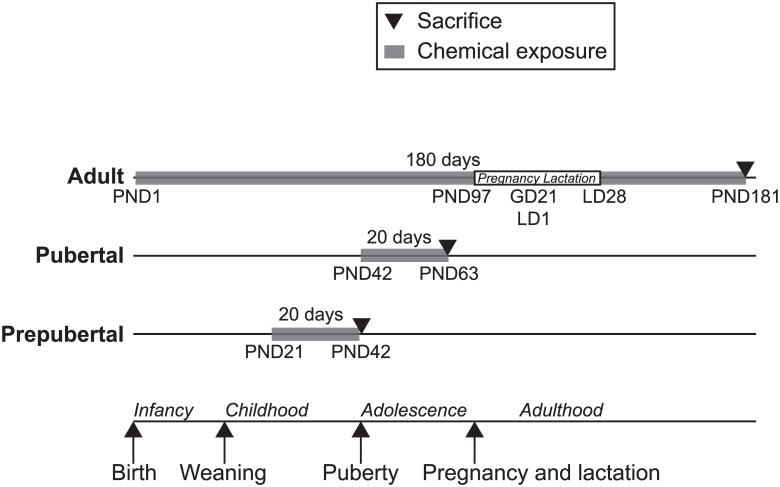
Schematic representation of the animal study. The adult group includes parous and nulliparous rats in equal numbers. Therefore the total number of rats in the adult group was twice the number of rats in the prepubertal and pubertal groups. PND denotes postnatal day. Adult rats were pregnant at PND97 with an average of 21 gestation days (GD) and 28 lactating days (LD) from delivery to weaning of the pups.

The animal experiment was carried out at the animal facilities of Ramazzini Institute (Bologna, Italy). The protocols were approved by the IACUC of both the Ramazzini Institute and Icahn School of Medicine at Mount Sinai. Because the current study was part of a parent study on the effect of environmental chemicals on breast cancer, only female Sprague-Dawley rats (Ramazzini Institute—Cesare Maltoni Cancer Research Center colony) were included. Rats were kept in makrolon cages (W16”xD10”xH6”) with a shallow layer of white wood shavings as bedding, a stainless steel wire mesh cover, and 2 or 3 animals per cage. Animals were housed in room specifically devoted to this study with access restricted to authorized personnel. Temperature was constant temperature at 22°C (± 3°C) and relative humidity was between 40 and 60%. Light was provided by artificial lamps with a light/dark cycle of 12 h/day. Occasional variations from the standard settings were recorded. Rats were fed with standard diet matching all international parameters, in pellet chow (Laboratorio Dottori Piccioni, Gessate MI, Italy). Water and food consumption, body weight and any clinical observations were recorded weekly for the first 13 weeks of the experiment and every other week thereafter. The doses for each chemical were determined through an exposure experiment conducted to identify the oral dose of each chemical that produced urinary biomarker concentrations that were in the range of the US population [23]. These doses (in mg/kg, DEP: 0.1735, MPB: 0.105, TCS: 0.05) are 10,000 fold lower than the reported NOAEL levels for DEP and MPB and 1,000 fold lower than the reported NOAEL levels for TCS [23, 24]. The timing of treatment and sample collection are illustrated in Fig 1. There are 5 treatment conditions (control, DEP, MPB, TCS and their mixture (Mix)) and three experimental groups of rats defined by windows of susceptibility, i.e. prepubertal and pubertal and chronic exposure from birth to adulthood (referred to as adult). The prepubertal group consisted of 25 rats with 5 rats for each exposure. The animals were exposed from PND21 to PND28 through the breast milk of daily gavaged lactating dams (F0). Subsequently, these rats were exposed by daily oral gavage from PND28 (weaning) to PND41 and sacrificed at PND42. The pubertal group consisted of 25 rats with 5 rats for each treatment group. The pubertal rats were exposed by daily oral gavage from PND42 to PND62 and sacrificed at PND63. The adult experimental group included 50 rats with 10 animals (5 parous and 5 nulliparous) for each treatment group. The animals were treated daily from PND1 to PND28 through milk of the lactating dams (F0). After weaning (PND28), the female offspring (F1) was treated through oral gavage 3 times a week (Monday, Wednesday and Friday) until the sacrifice at PND181 [24]. DEP, MPB and TCS were supplied by Sigma Aldrich (Milan, Italy). Olive Oil (Montalbano Agricola Alimentare Toscana, Florence, Italy) was used as vehicle to prepare all dosing solutions. During the experiment, each compound was stored in the dark at room temperature (20°C). The solutions were prepared the first day of treatment and used for the whole duration of the experiment. The control group was treated with olive oil only. To minimize external contamination, the olive oil and chemicals were stored in glass containers and administered using 5mL glass syringes. DEP, MPB and TCS were not detected in the olive oil used as vehicle using GC/MS (Neotron Laboratory, Modena, Italy). Chemicals were administered by gastric intubation (gavage) starting with 0.5 mL/animal/dose from PND28 to PND63, and then with 1 mL/animal/dose until PND181. All animals were sacrificed in the morning by carbon dioxide and necropsy was immediately performed for the collection of selected tissues. All animals belonging to the same developmental group were sacrificed on the same day. The estrous stage of the animals was unknown. Whole blood was collected from the vena cava inferior directly after sacrifice. Adult rats were sacrificed at PND181, which for the parous rats was 1 month after the end of the pregnancy and lactation period. The cryovials were flash-frozen in liquid nitrogen and stored at -80°C.

### Non-Targeted Metabolomic Profiling

Metabolomic profiling was performed on whole blood samples. Plasma, serum or urine was not available. Samples were shipped to Metabolon (Durham, NC) on dry ice and analyzed according to their standard protocol. The non-targeted metabolic profiling pipeline combined four independent platforms: ultra-high performance liquid chromatography/tandem mass spectrometry (UHPLC/MS/MS) optimized for basic species, UHPLC/MS/MS optimized for acidic species, UHPLC/MS/MS optimized for polar species, and gas chromatography/mass spectrometry (GC/MS). Samples were processed essentially as described previously [25–27]. For each sample, 100μL of whole blood was used for analyses. Using an automated liquid handler (Hamilton LabStar, Salt Lake City, UT), protein was precipitated with methanol that contained standards to report on extraction efficiency. The resulting supernatant was split into five aliquots for analysis on the four platforms, with one aliquot retained as a spare. Aliquots were dried under nitrogen, vacuum-desiccated and subsequently reconstituted. For the UHPLC/MS/MS analyses, samples were dissolved either in 50μL 0.1% formic acid in water (acidic conditions), or in 50μL 6.5mM ammonium bicarbonate in water, pH 8 (basic conditions). For GC/MS analysis, samples were derivatized at 60°C for one hour in a final volume of 50μL using equal parts bistrimethyl-silyl-trifluoroacetamide and solvent mixture acetonitrile:dichloromethane:cyclohexane (5:4:1) with 5% triethylamine. In addition, three types of controls were analyzed in concert with the experimental samples: aliquots of a “client matrix” formed by pooling a small amount of each sample served as technical replicates throughout the data set, extracted water samples served as process blanks, and a cocktail of standards spiked into every analyzed sample allowed instrument performance monitoring. Experimental samples and controls were randomized across three platform run days.

For UHLC/MS/MS analysis, aliquots were separated using a Waters Acquity UPLC (Waters, Millford, MA) and analyzed using a Q-Exactive high resolution/accurate mass spectrometer (Thermo Fisher Scientific, Inc., Waltham, MA) which consisted of an electrospray ionization (ESI) source and Orbitrap mass analyzer. Derivatized samples for GC/MS were separated on a 5% phenyldimethyl silicone column with helium as the carrier gas and a temperature ramp from 60°C to 340°C and then analyzed on a Thermo-Finnigan Trace DSQ MS (Thermo Fisher Scientific, Inc.) operated at unit mass resolving power with electron impact ionization and a 50–750 atomic mass unit scan range.

Metabolites were identified by automated comparison of the ion features in the experimental samples to a reference library of chemical standard entries that included retention time, molecular weight (m/z), preferred adducts, and in-source fragments as well as associated MS spectra, and were curated by visual inspection for quality control using software developed at Metabolon [28].

### Quantification of Sulfonated Steroids

Whole blood samples were shipped to Steroid Laboratory of the Children’s Hospital Giessen (Germany) on dry ice for quantification of cholesterol sulfate, androgen sulfates, and progestagen sulfates by LC-MS/MS essentially as described [29]. We were able to quantify cholesterol sulfate, pregnenolone sulfate, 17-hydroxy-pregnenolone sulfate, androsterone sulfate and epiandrosterone sulfate. Levels of other sulfonated steroids were below the limit of quantification.

### Statistical Analysis

We used the MetaboAnalyst version 2.5 for statistical analysis [30]. A total of 591 distinct metabolites were detected in the blood samples (S1 Table). Some metabolites were not detected in all samples (total of 1947 missing values; 3.3%). Up to 36 metabolites were removed because they were not detected in more than 30% of the samples of each developmental window (S1 Table). These 36 metabolites did not show an obvious correlation with any of the exposures. The remaining missing variables were replaced with half of the minimum value for each feature column. We employed no data filtering. Next the data were scaled as mean-centered and divided by the standard deviation of each variable (z score). Adjustment for developmental stage was performed by expressing each metabolite as its z score for the three developmental stages separately after which the data for all groups were combined again effectively reducing the variation introduced by the treatment windows. Differentially expressed metabolites between the five groups were determined using the Kruskal-Wallis non parametric ANOVA using a false discovery rate (FDR) adjusted (Benjamini-Hochberg) p value (q) cutoff of 0.1. The Post Hoc multiple comparisons were performed using SPSS version 20 (IBM). For this we used Tamhane’s T2, which does not assume equal variances between groups.

## Results

### Pronounced Changes in the Rat Metabolome during Development

**Fig 2:**
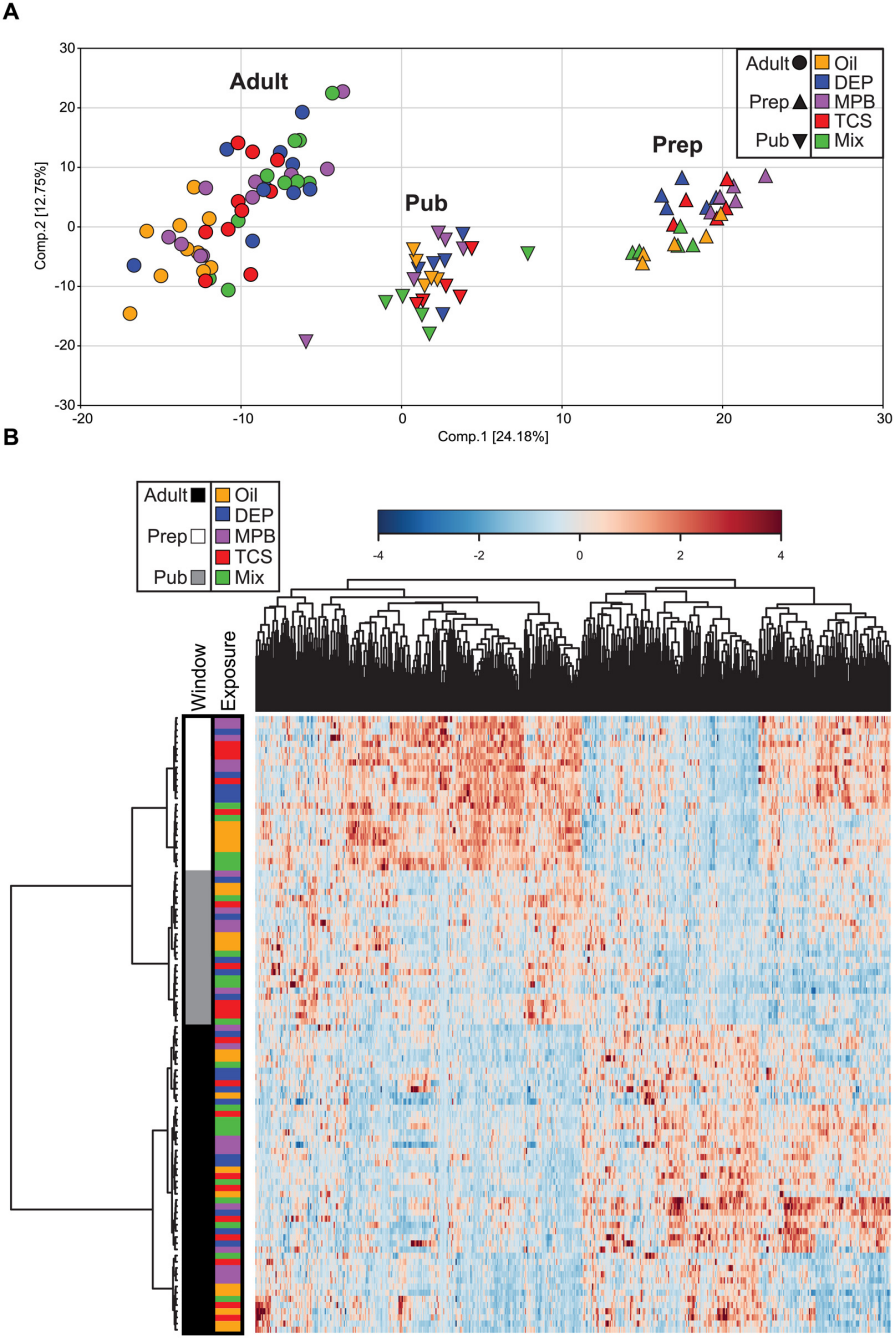
Characterization of the metabolomic dataset. (A) Principal component analysis showing the first and second component, and the separation of all samples. (B) Heatmap and hierarchical clustering of all samples. The distance measure is Spearman and the clustering algorithm is Ward. The samples are colored by age group and treatment.

Non-targeted metabolomic profiles were determined in whole blood collected from female rats that were exposed to three different personal care product ingredients (DEP, MPB, and TCS) or their mixture with three different treatment periods during development: prepubertal, pubertal, birth to adulthood (adult). Overall intermediary metabolism is comparable between rats and humans as at least 86% of the detectable 591 metabolites have a Human Metabolome Database ID [31] with the remaining metabolites being either not annotated yet or in exceptional cases not human. A principle component analysis showed that most of the variation in the metabolites was associated with developmental stage with PC1 and PC2 separating prepubertal, pubertal and adult rats (Fig 2A). Most of the consistently detected metabolites showed significant changes across developmental windows regardless of treatment (S2 Table). This is clearly illustrated by a heatmap visualizing all analyzed metabolites, which clusters the animals by developmental window (Fig 2B). Similar results were obtained when restricting analyses to control animals only. As expected, these results indicate that development itself has a strong impact on the blood metabolome profile.

### Low-Dose Exposure to Environmental Chemicals Affects the Metabolome

The principal component analysis indicated that there is little variation in the metabolome profiles associated with environmental chemical exposure (Fig 2A). Visualization of the treatment groups in the heatmap of the complete dataset shows the relatively small impact of the different chemical exposures on metabolism when compared to developmental age (Fig 2B).

**Fig 3:**
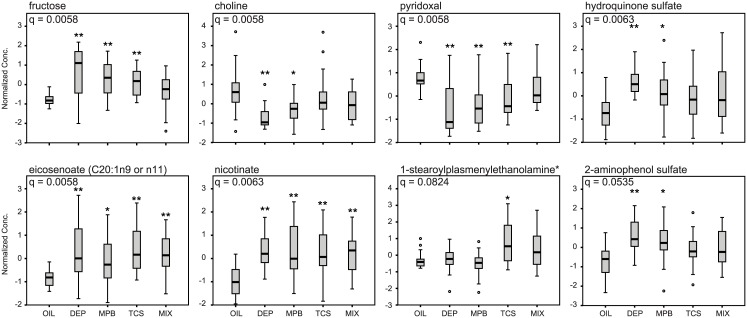
Selected metabolites changed by exposure to personal care product ingredients. Box and whisker plots of eight selected metabolites of the group of 42 metabolites that were significantly changed by exposure to personal care product ingredients regardless of treatment window. The FDR (q) is indicated for each metabolite. The significance of the post hoc comparison of the treatment groups to oil is indicated as follows: ** P < 0.01 or * P < 0.05.

Given that this dataset contains a high number of variables with a relatively low sample size per group, we first analyzed the effect of exposure to DEP, MPB, TCS and Mix in the complete cohort of rats irrespective of treatment window. We identified 42 metabolites that were affected significantly by the treatment (S3 Table, “short list”, q < 0.1). The top 8 metabolites on this list were also significant with a more conservative q (S3 Table, < 0.05). Using hierarchical clustering, this set of 42 metabolites did not group the animals according to the different exposures, although most of the untreated control animals tended to cluster (S1 Fig). The most significantly changed metabolite was fructose, which was elevated in all treatment groups (except for the Mix group) compared to the controls (Fig 3). Seven of the affected metabolites were unsaturated fatty acids, mostly polyunsaturated of the n-6 series, which in general increased under all treatment conditions (Fig 3 and S1 Fig). Seven other changed metabolites were aromatic sulfate conjugates, which significantly increased with DEP and MPB (Fig 3 and S1 Fig). The unsaturated fatty acids, but also the aromatic sulfate conjugates correlated with each other, which is illustrated by the clustering in the heatmap (S1 Fig). This indicates that these metabolites respond similarly to the different exposures. Levels of serotonin and its breakdown product 5-hydroxyindoleacetic acid were also clustered in the heatmap. Changes were most pronounced for 5-hydroxyindoleacetic acid, which significantly increased in all treated groups including the mixture (S1 Fig). Pyridoxal (vitamin B6) levels were decreased in response to the individual exposures, while its levels were unaffected in the Mix group. In contrast, levels of nicotinate (vitamin B3) increased in response to exposure (Fig 3). Two lyso-plasmalogens appeared to be specifically increased by TCS (Fig 3 and S1 Fig). Other affected metabolites included sphinganine, choline and ethanolamine (Fig 3 and S1 Fig). These results illustrate that exposure to personal care product ingredients leads to significant changes in the blood metabolome in multiple biochemical pathways.

Since development itself had a major impact on the blood metabolome, we re-examined the chemical-specific effects after centering the metabolite values around the mean for the prepubertal, pubertal and adult groups separately and then combining all values again. This procedure effectively reduced the variation introduced by the different developmental stages and resulted in a significant increase in the number of metabolites that were modified by exposure to the chemicals (162 metabolites with q < 0.1, S4 Table). All 42 metabolites on the short list were also represented in this extended list. Of these, 41 had improved q values, which are all < 0.05 and varied between 0.025 and 1.6 x 10^−6^ (S4 Table) showing that the analysis is robust. The most significantly altered metabolite in this analysis was 5-oxoproline for which the FDR decreased from 0.11407 to 1.4 x 10^−6^. Other metabolites that entered the top 20 of most affected metabolites were adenosine-5'-diphosphoglucose, 2'-deoxyuridine, 4-hydroxybutyrate (GHB), 2,3-diphosphoglycerate, trigonelline (N'-methylnicotinate), inosine 5'-monophosphate (IMP) and sphingosine (S4 Table). Most of these metabolites were significantly affected by development masking the effect of exposure. Collectively, these results further demonstrate that exposure to personal care product ingredients is capable of inducing measurable changes in global metabolism, even at very low doses.

### Window-Specific Changes in the Metabolome by Environmental Chemicals

**Fig 4:**
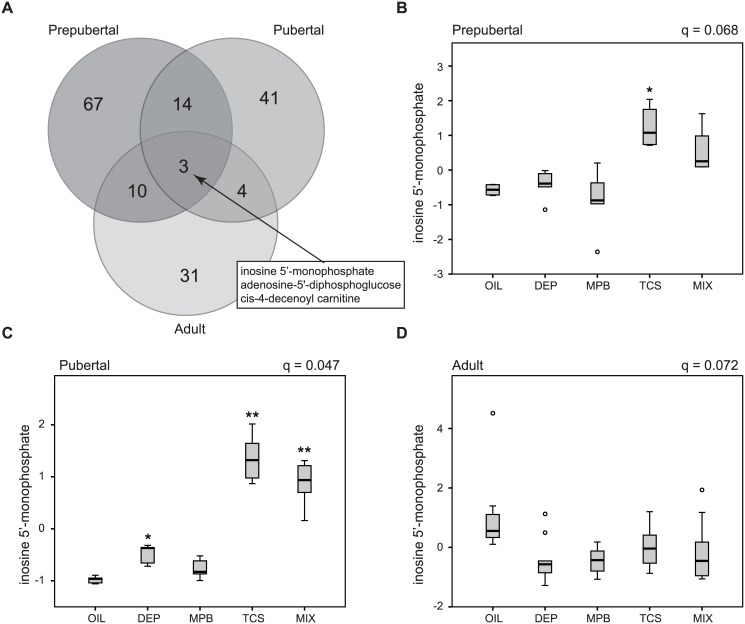
Different sensitivity to personal care product ingredients at the different treatment windows. (A) Venn diagrams showing overlap in the metabolites that were significantly affected at the three different treatment windows. Inosine 5’-monophosphate levels at the prepubertal (B), pubertal (C) and adult stage (D). The FDR (q) is indicated for each treatment window. The significance of the post hoc comparison of the treatment groups to oil is indicated as follows: ** P < 0.01 or * P < 0.05.

In order to assess whether the sensitivity to environmental insults varies at different developmental windows, we analyzed the data in each of the three developmental windows independently. We identified 95 significantly changed metabolites (q < 0.1) in the prepubertal group (S5 Table), 62 in the pubertal group (S6 Table) and 48 in the adult group (S7 Table). No differentially expressed metabolites were identified when parous and nulliparous (adult) animals were analyzed separately. Although none of the metabolites in the prepubertal group were changed at a more conservative cutoff (q < 0.05, S5 Table), 26 in the pubertal group (S6 Table) and 18 in the adult group (S7 Table) remained significant. An intersection between the three developmental stage-specific metabolite sets (at a q < 0.1) showed that a total of 170 metabolites were affected by exposure, 18% of which (31 out of 170) was changed in at least two treatment windows with only 3 of those metabolites affected in all windows (Fig 4A and S8 Table). Of these 31 metabolites, 8 (26%) were also found in the short list of metabolites that changed regardless of treatment window and 26 (84%) were present in the extended list of affected metabolites after correction for developmental window (S8 Table). As such these are the metabolites consistently identified as affected by the different exposures. Interestingly, 12 metabolites (29%) on the short list were not significantly changed in any of the three treatment windows alone indicating that they have a relatively small change that is only detectable in a larger group of animals. Combined these observations further demonstrate that exposure to personal care product ingredients leads to significant metabolic changes regardless of age of exposure.

Three metabolites, inosine 5'-monophosphate (inosinic acid or IMP), adenosine-5'-diphosphoglucose and cis-4-decenoyl carnitine were affected in all developmental windows. Interestingly, IMP and adenosine-5'-diphosphoglucose did not uniformly respond to the exposure at each age group. While TCS and Mix increased both metabolites in the prepubertal and pubertal stage, there was an overall decrease upon treatment in the adult group (Fig 4B–4D for IMP). This indicates that metabolites can be differentially affected by personal care product ingredient exposure at different treatment windows.

**Fig 5:**
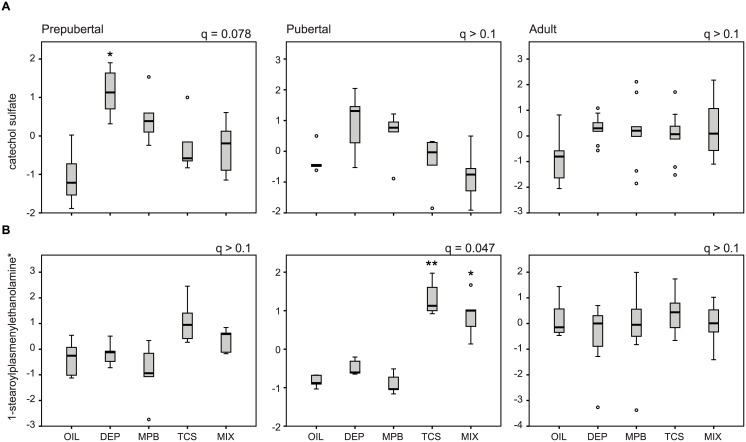
Metabolites are differentially affected by personal care product ingredients exposure at different treatment windows. Levels of catechol sulfate (A) and 1-stearoylplasmenylethanolamine (B) at the prepubertal, pubertal and adult stage. The FDR (q) is indicated for each treatment window. The significance of the post hoc comparison of the treatment groups to oil is indicated as follows: ** P < 0.01 or * P < 0.05.

Moreover, changes in 82% of the metabolites were only observed in one specific treatment window (139 out of 170). Remarkably 16% of these metabolites were also found in the short list of metabolites that changed regardless of treatment window (22 out of 139). This indicates that these metabolites were strongly affected at one particular treatment window, with more modest or no response at the other developmental stages. These metabolites included many of the aromatic sulfate conjugates such as hydroquinone sulfate 2-aminophenol sulfate, O-methylcatechol sulfate and catechol sulfate, which were most significantly altered in the prepubertal stage and showed less pronounced differences in the pubertal and adult stage (Fig 5A for catechol sulfate). Changes in the lyso-plasmalogens upon TCS exposure were most significant in the pubertal stage with no change in the adult stage (Fig 5B). All other metabolites (117) were uniquely changed in one developmental window. This illustrates that these metabolites are differentially affected by personal care product ingredient exposure at different treatment windows.

### Phenol Sulfonation Is Affected by Environmental Chemicals

**Fig 6:**
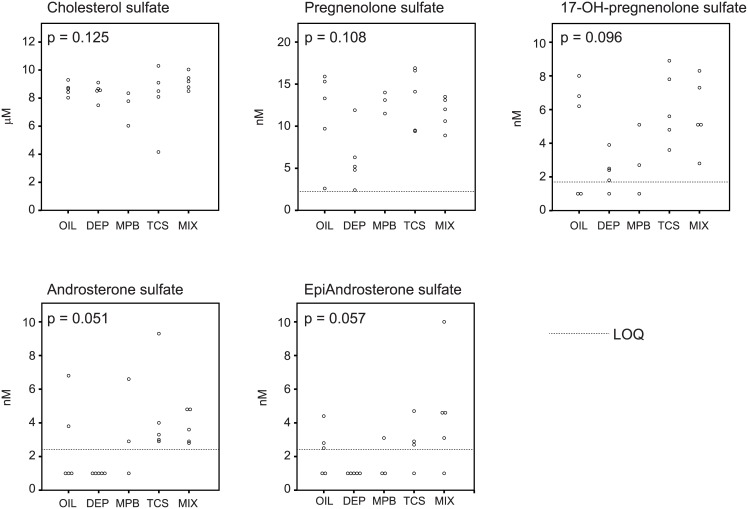
Cholesterol sulfate and sulfonated steroids. Dot plots of cholesterol sulfate, pregnenolone sulfate (5-pregnen-3β-ol-20-one-3-sulfate), 17-hydroxy-pregnenolone sulfate, androsterone sulfate and epiandrosterone sulfate in the prepubertal group. LOQ stand for limit of quantification. For statistical analysis, samples with values < LOQ were set at 1 nM. The indicated P values are the result of the Kruskal-Wallis non parametric ANOVA. For 2 rats in the MBP group, we did not have sufficient sample for the analysis.

We observed a significant increase in aromatic sulfate conjugates in response to treatment with DEP in the prepubertal stage (S8 Table and Fig 5A). The levels of aromatic sulfate conjugates are highly correlated in the prepubertal animals. In contrast the levels of inorganic sulfate were lower in response to treatment with DEP (q = 0.070). In accordance, the levels of catechol sulfate correlated inversely with inorganic sulfate levels (Table 1). To test if these findings signified a more general perturbation of sulfate conjugation, we quantified cholesterol sulfate, pregnenolone sulfate, 17-hydroxy-pregnenolone sulfate, androsterone sulfate and epiandrosterone sulfate in the blood samples of the prepubertal rats. Blood cholesterol sulfate levels were similar among all prepubertal groups (Fig 6). The levels of all four sulfonated steroids were lowest in the DEP-treated rats and frequently below the level of quantification (Fig 6). These differences, however, did not reach statistical significance (Fig 6).

**Table 1:** Correlation coefficient between 2-aminophenol sulfate, catechol sulfate, hydroquinone sulfate, O-methylcatechol sulfate and sulfate in the prepubertal stage.

	2-aminophenol sulfate	catechol sulfate	hydroquinone sulfate	O-methylcatechol sulfate	sulfate
2-aminophenol sulfate	1	0.936 **	0.889 **	0.935 **	-0.250
catechol sulfate	0.944 **	1	0.819 **	0.950 **	-0.293
hydroquinone sulfate	0.884 **	0.837 **	1	0.836 **	-0.130
O-methylcatechol sulfate	0.949 **	0.958 **	0.861 **	1	-0.145
sulfate	-0.387	-0.443 *	-0.309	-0.345	1

The Pearson correlation coefficient is provided above the diagonal, Spearman’s rho below. The coefficients were calculated using all 25 samples.

** denotes P<0.01,

* denotes P<0.05.

### Pyruvate Metabolism Is Affected by Environmental Chemicals

**Fig 7:**
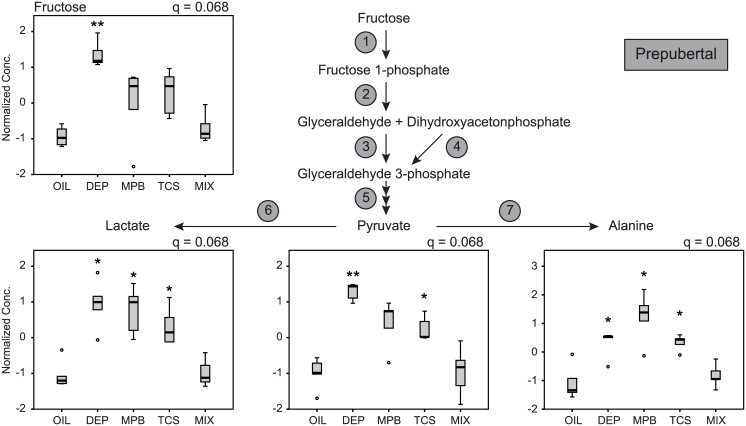
Fructose and pyruvate metabolism. Box and whisker plots of fructose, pyruvate, lactate and alanine in the prepubertal group. Given the tight correlation between these metabolites, we displayed the pyruvate metabolic pathway with its intermediates and enzymes. Enzyme 1, Fructokinase; 2, Aldolase B; 3, Glyceraldehyde kinase; 4, Triose phosphate isomerase; 5, further classic glycolytic enzymes; 6, Lactate dehydrogenase; 7, alanine transaminase. The FDR (q) is indicated for each metabolite. The significance of the post hoc comparison of the treatment groups to oil is indicated as follows: ** P < 0.01 or * P < 0.05.

Since pyruvate is an important metabolite in intermediary metabolism, we further explored the changes in its levels by exposure to personal care product ingredients. Correction for developmental window decreased the FDR from 0.054 to 1.6 x 10^−6^ making pyruvate the second most significantly affected metabolite after 5-oxoproline (S4 Table). Since pyruvate is an unstable metabolite, we first assessed the quality of our pyruvate measurement. Pyruvate is in equilibrium with lactate and alanine via lactate dehydrogenase and alanine transaminase respectively (Fig 7). We observed a high correlation coefficient between levels of pyruvate, lactate and alanine illustrating the validity of these measures (S9 Table). Pyruvate levels also closely correlated with fructose levels suggesting that these metabolites are metabolically related (S9 Table). Next we analyzed the effect of exposure on pyruvate metabolism at each developmental window. Most pronounced changes were observed in the prepubertal stage, with all pyruvate-related metabolites increased by the individual personal care product ingredients (Fig 7). Remarkably, the Mix treatment reversed these changes. Similar changes but only significant for fructose and pyruvate were observed in the adult stage (S2 Fig). In the pubertal stage none of these metabolites had and FDR < 0.1 (S3 Fig). Thus these results indicate that personal care product ingredients affect pyruvate metabolism, but in a developmental window-dependent fashion.

## Discussion

We investigated the effects of low-dose exposure to environmental chemicals commonly used in personal care products on the blood metabolome at three different windows of susceptibility in a rat model. The rats were treated at levels comparable to human exposures, which are 1,000–10,000 fold lower than the NOAEL and much lower than most doses used in published studies [23]. Therefore, this study was not designed to study cause and effect for toxicity or major phenotypic changes, but rather to detect biochemical signature changes that reflect human exposure scenarios. Our results show that most of the variation in the metabolites was associated with developmental stage. The low-dose exposure to DEP, MPB and TCS had a relatively small, but detectable impact on the metabolome. We note that multiple metabolites altered by exposure belong to the same biochemical pathways, including phenol sulfonation, and metabolism of pyruvate, lyso-plasmalogens, unsaturated fatty acids and serotonin, implicating potential mechanistic pathways that these ingredients may influence. We further illustrate that these changes in the metabolome differed by developmental age.

It is well established that the natural process of growth and development occurring from childhood through adulthood results in notable metabolic changes. In clinical chemistry it is therefore common practice to use age-specific ranges for most parameters. This is clearly reflected in our dataset in which development itself had a strong impact on the rat blood metabolome. Our study results clearly demonstrate that susceptibility to personal care product ingredient exposure differs with respect to developmental stage, supporting the concept of “windows of susceptibility”. The highest number of affected metabolites was observed in the prepubertal stage while slightly fewer metabolites were changed at the pubertal stage. Remarkably rats that underwent chronic exposure from birth to adulthood displayed the smallest number of changes. In fact, the changes in the blood metabolome identified after chronic exposure were only found to be statistically significant when the parous and nulliparous subgroups were collapsed. The parous rats were sacrificed approximately 1 month after pregnancy and lactation likely minimizing differences with the nulliparous group. Therefore it appears that chronic exposure may lead to long-term adaptations that are not evident after shorter term exposure at the prepubertal and pubertal windows. The time prior to pubertal development has been identified as a window of susceptibility for chronic diseases including breast cancer [32–34]. Our experimental observation meshes well with the findings in humans and suggests that the prepubertal stage is the most sensitive window of opportunity for these personal care product ingredients.

Multiple aromatic sulfate conjugates were affected by exposure in the prepubertal stage, most significantly by DEP. These include 2-aminophenol sulfate, a biomarker for whole grain rye bread consumption [35], and sulfate esters of hydroquinone and catechol, benzene-1,4-diol and benzene-1,2-diol. Hydroquinone and catechol are metabolites in benzene metabolism and involved in its toxicity [36, 37], but have other sources as well. Conjugation with sulfate is an important phase II pathway for the biotransformation of these aromatic compounds. Our data indicate that DEP impacts on and possibly induces this pathway leading to increased sulfate conjugation of environmental aromatic compounds. The induction of sulfate conjugation may not be a completely unexpected as all tested personal care product ingredients are aromatic compounds as well. Indeed although sulfotransferase activity may be inhibited upon exposure to bisphenol A and TCS [38], their transcription is regulated by a variety of transcription factors including xenobiotic receptors [39, 40]. TCS has been reported to activate human and rodent xenobiotic nuclear receptors [41, 42], whereas DEP had only little effect on these receptors [43]. No studies report on the effect of MPB on xenobiotic metabolism.

Importantly, our data suggest that low-dose exposure to DEP in the prepubertal stage may lead to depletion of inorganic sulfate. We found that in prepubertal rats exposure not only changed the levels of sulfonated phenols but also sulfate itself. Indeed levels of sulfate and catechol sulfate were inversely correlated. Systemic depletion of inorganic sulfate secondary to utilization of this anion for the sulfonation of specific environmental chemicals may therefore affect the availability of sulfate for other (endogenous) substances that are subject to sulfate conjugation such as steroids, proteoglycan and lipids [44]. Sulfonation plays a critical role in the transport and biosynthesis of steroid hormones. Sulfate esters of dehydroepiandrosterone, 17β-estradiol and estrone are transport forms of these steroids and are intermediates in androgen and estrogen biosynthesis [38]. Indeed estrone sulfate is a potential source of estradiol in human breast cancer tissues [45]. Moreover, urinary phthalates have been associated with adrenal androgen levels including dehydroepiandrostenedione sulfate [46]. In order to test such a mechanistic link, we analyzed levels of cholesterol sulfate, androgen sulfates, and progestagen sulfates in the blood of all prepubertal rats. Although not significant for the individual sulfonated steroids, they were all 4 consistently lower in DEP-treated animals. Therefore we feel that this combination of perturbations in sulfonation by DEP warrants further investigation.

We showed a pronounced effect of environmental chemicals on pyruvate metabolism at the prepubertal stage. Exposure not only increased pyruvate levels, but also many related metabolites such as alanine and lactate as well as phenylpyruvate. In clinical biochemistry, accumulation of these metabolites indicates a defect in pyruvate metabolism which occurs in several mitochondrial diseases. The origin of the pyruvate increase in our study is unclear as it is unlikely that the exposures would have caused a significant defect in mitochondrial metabolism. We did not find evidence that these changes in pyruvate concentration were related to changes in food intake or bodyweight. Pyruvate levels were also closely correlated to fructose levels. Although pyruvate is a product of fructose degradation (Fig 6), the reason for this correlation is unclear as glucose is considered the major source of pyruvate (and alanine) under regular conditions. DEP, MPB and TCS have not been associated yet with changes in pyruvate metabolism. Future studies should be aimed at identifying the cause of the pyruvate increase, which could be addressed with metabolic flux studies.

Our analysis also revealed that serotonin and its metabolite 5-hydroxyindoleacetic acid were increased by all personal care product ingredients. Peripheral serotonin levels are mainly determined by its release from activate platelets and degradation to 5-hydroxyindoleacetic acid by monoamine oxidase A (MAO) [47]. This observation suggests that environmental chemicals can activate platelets or inhibit MAO, but there are no studies available that investigated this link.

TCS increased several lyso-plasmalogen species. Plasmalogens are phospholipids containing an acid labile vinyl ether group at the *sn*-1 position. They have multiple functions including serving as endogenous antioxidant, mediators of biological membranes and storage for polyunsaturated fatty acids [48, 49]. Lyso-plasmalogens are the product of phospholipase A2 and are further processed by lysoplasmalogenase [48]. So far one study has found that TCS interferes with phospholipase A2 and as such can reduce prostaglandin biosynthesis in human gingival fibroblasts [50].

The most significantly altered metabolite after adjustment for developmental window was 5-oxoproline (pyroglutamic acid), which is an intermediate in the gamma-glutamyl cycle. Four other intermediates from this cycle are significantly altered by exposure after age correction, gamma-glutamylalanine, oxidized glutathione, gamma-glutamylthreonine and gamma-glutamylcysteine (S4 Table). Of these, gamma-glutamylalanine and gamma-glutamylthreonine are negatively correlated with 5-oxoproline (Spearman’s rho -0.368 and -0.298, respectively, P < 0.01). The gamma-glutamyl cycle plays a role in glutathione metabolism and possibly amino acid membrane transport [51].

One of the highlights of our investigation is the attempt to study the consequences of exposure to a mixture of personal care product ingredients. This is an understudied area of environmental research although a recent initiative aims to characterize the combined effects of mixtures of pesticide residues [52]. Different biological activities have been described for DEP, MPB and TCS. Therefore, there was no *a priori* assumption of additive, synergistic or antagonistic interactions. Indeed, for some metabolites the response in the mix group was comparable to the response to one of the individual chemicals, such as the increases of 1-stearoylplasmenylethanolamine, IMP and adenosine-5'-diphosphoglucose in the TCS and Mix group. For some other metabolites however, we observed antagonistic effects of individual ingredients as animals treated with the mixture produced much less prominent changes compared to those treated with individual personal care product ingredients. One such example is the minimization of the DEP-induced changes in aromatic sulfate conjugates, pyruvate and fructose in the Mix group. These data suggest that interactions of the individual personal care product ingredients can occur in multiple ways and should be further investigated.

The unique approach of investigating the metabolome after exposure to personal care product ingredients has revealed metabolic pathways that are affected at specific windows of susceptibility. The doses used in these experiments were chosen to mimic real human exposure levels [23]. The fact that we observed clear changes in the metabolome associated with these low dose exposures provides another indication that concern about health effects associated with exposure to these environmental chemicals is warranted. Potential limitations of our study are the lack of disease endpoints, the small number of animals per group and the absence of a replication experiment. Although it is not uncommon to use whole blood as the matrix for metabolite analysis, it makes a direct comparison with plasma metabolite levels less straightforward as some of the observed changes may be restricted to red blood cells or other cell types.

## Conclusions

Untargeted metabolite profiling revealed that exposure to environmental chemicals commonly used in personal care products can affect metabolism at very low doses. These metabolic responses vary depending on chemical and developmental age. Detailed analysis of the affected metabolites and biochemical pathways has highlighted potential mechanisms of action that warrant further investigation.

## Supporting Information

S1 Fig Heatmap and hierarchical clustering of the 42 metabolites that were significantly changed by exposure to endocrine disruptors regardless of treatment window. The distance measure is Spearman and the clustering algorithm is Ward. Clusters of metabolites are indicated by a bar and names in bold letters.
(PDF)

S2 Fig Box and whisker plots of fructose, pyruvate, lactate and alanine in the adult group. The significance of the post hoc comparison of the treatment groups to oil is indicated as follows: ** P < 0.01 or * P < 0.05.
(PDF)

S3 Fig Box and whisker plots of fructose, pyruvate, lactate and alanine in the pubertal group. The significance of the post hoc comparison of the treatment groups to oil is indicated as follows: ** P < 0.01 or * P < 0.05.
(PDF)

S1 Table Complete dataset as provided by Metabolon. (XLSX)

S2 Table Metabolites significantly changed at the different developmental windows. (XLSX)

S3 Table A “short list” of metabolites significantly changed by exposure to personal care product ingredients. (XLSX)

S4 Table Metabolites significantly changed by exposure to endocrine disruptors after correction for developmental window. (XLSX)

S5 Table Metabolites significantly changed by exposure to endocrine disruptors at the prepubertal developmental window. (XLSX)

S6 Table Metabolites significantly changed by exposure to endocrine disruptors at the pubertal developmental window. (XLSX)

S7 Table Metabolites significantly changed by exposure to endocrine disruptors at the adult developmental window. (XLSX)

S8 Table An intersection between the significantly changed metabolites after exposure to endocrine disruptors at the three different developmental windows. (XLSX)

S9 Table Correlation coefficient between alanine, pyruvate, lactate and fructose. (XLSX)
