# Supplementary figures and images for: Changes in the Metabolome in Response to Low-Dose Exposure to Environmental Chemicals Used in Personal Care Products during Different Windows of Susceptibility
Source: PLoS One. 2016 Jul 28;11(7):e0159919. doi: 10.1371/journal.pone.0159919 (PMC4965097; doi:10.1371/journal.pone.0159919)

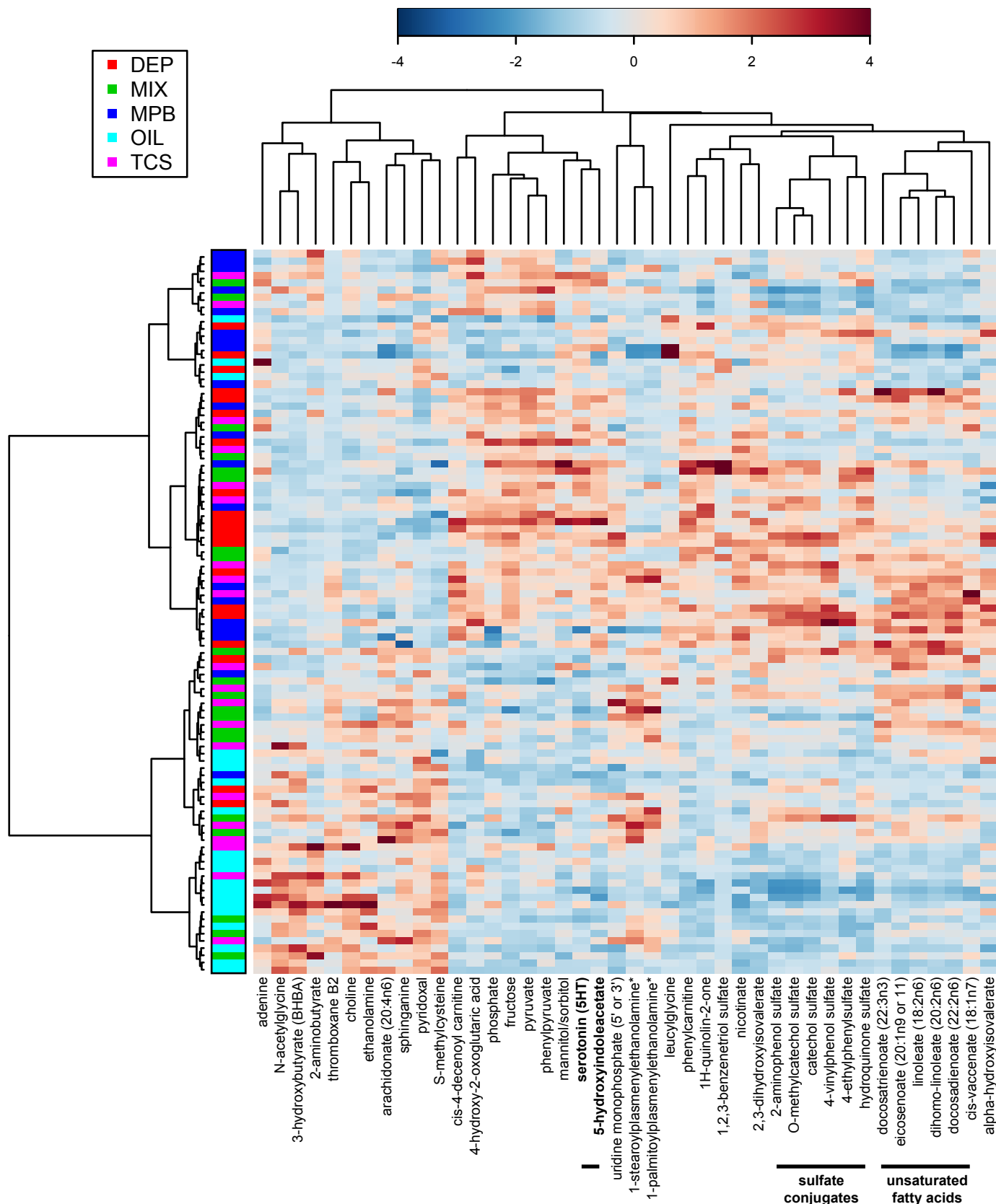

Supplement: S1 Fig — The distance measure is Spearman and the clustering algorithm is Ward. Clusters of metabolites are indicated by a bar and names in bold letters. (PDF) [file pone.0159919.s001.pdf]

Adult

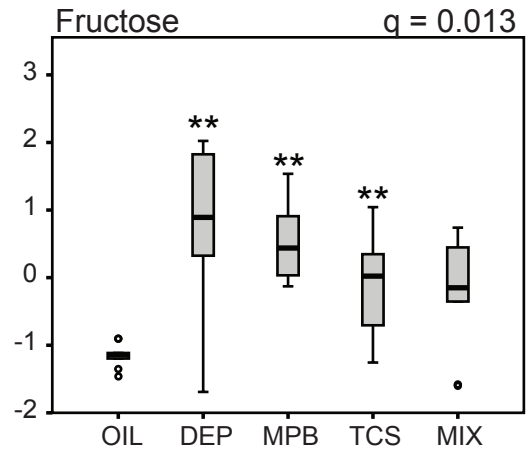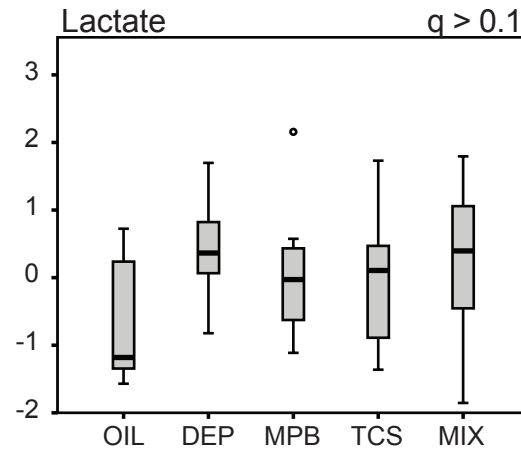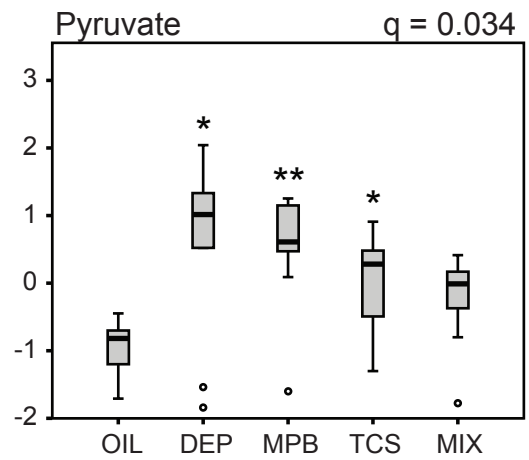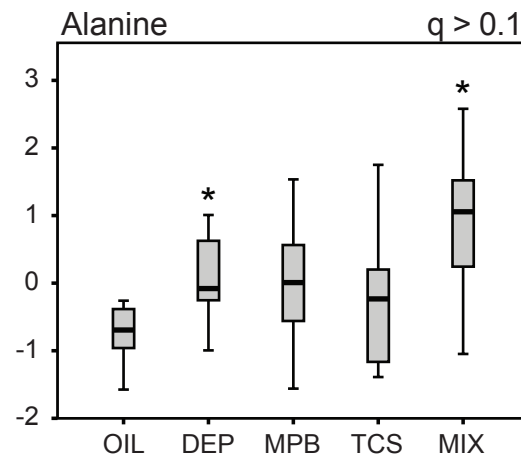

Supplement: S2 Fig — The significance of the post hoc comparison of the treatment groups to oil is indicated as follows: ** P < 0.01 or * P < 0.05. (PDF) [file pone.0159919.s002.pdf]

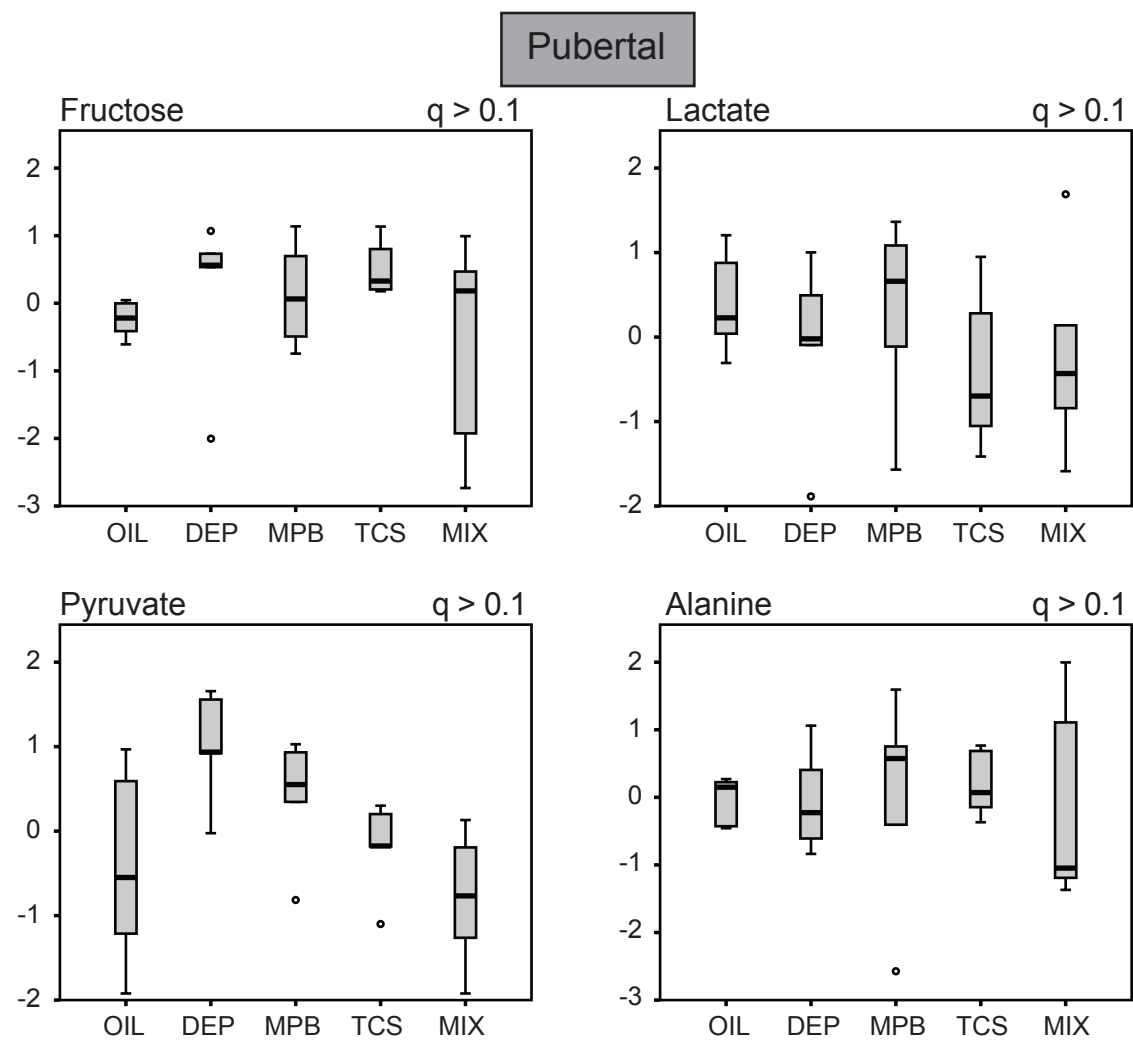

Supplement: S3 Fig — The significance of the post hoc comparison of the treatment groups to oil is indicated as follows: ** P < 0.01 or * P < 0.05. (PDF) [file pone.0159919.s003.pdf]
